# Social structure modulates the evolutionary consequences of social plasticity: A social network perspective on interacting phenotypes

**DOI:** 10.1002/ece3.3753

**Published:** 2017-12-27

**Authors:** Pierre‐Olivier Montiglio, Joel W. McGlothlin, Damien R. Farine

**Affiliations:** ^1^ Department of Biology & Redpath Museum McGill University Montreal QC Canada; ^2^ Department of Biological Sciences Virginia Tech Blacksburg VA USA; ^3^ Department of Collective Behaviour Max Planck Institute for Ornithology Konstanz Germany; ^4^ Department of Biology Chair of Biodiversity and Collective Behaviour University of Konstanz Konstanz Germany; ^5^ Department of Zoology Edward Grey Institute University of Oxford Oxford UK

**Keywords:** evolution, quantitative genetics, social interactions, social network, social plasticity

## Abstract

Organisms express phenotypic plasticity during social interactions. Interacting phenotype theory has explored the consequences of social plasticity for evolution, but it is unclear how this theory applies to complex social structures. We adapt interacting phenotype models to general social structures to explore how the number of social connections between individuals and preference for phenotypically similar social partners affect phenotypic variation and evolution. We derive an analytical model that ignores phenotypic feedback and use simulations to test the predictions of this model. We find that adapting previous models to more general social structures does not alter their general conclusions but generates insights into the effect of social plasticity and social structure on the maintenance of phenotypic variation and evolution. Contribution of indirect genetic effects to phenotypic variance is highest when interactions occur at intermediate densities and decrease at higher densities, when individuals approach interacting with all group members, homogenizing the social environment across individuals. However, evolutionary response to selection tends to increase at greater network densities as the effects of an individual's genes are amplified through increasing effects on other group members. Preferential associations among similar individuals (homophily) increase both phenotypic variance within groups and evolutionary response to selection. Our results represent a first step in relating social network structure to the expression of social plasticity and evolutionary responses to selection.

## 1 INTRODUCTION

Interactions among organisms are ubiquitous in nature. For example, individuals interact with conspecifics when acquiring or defending food, refuges, or mates (Clutton‐Brock, 1989; Giraldeau & Caraco, 2000; Huntingford & Turner, 1987; Krause & Ruxton, 2002), and with heterospecifics in mutualism, antagonism, and competition (e.g., Crowley & Cox, 2011; Miller, Ament, & Schmitz, 2014; Shuster, Lonsdorf, Wimp, Bailey, & Whitham, 2006; Thompson, 1982). In response to such interactions, individuals may adjust their phenotype as a function of the phenotype of those with which they interact (Fawcett & Johnstone, 2010; West‐Eberhard, 1989). For example, individuals might express stronger aggression in the presence of more aggressive individuals than in the presence of more passive individuals (Wilson, Gelin, Perron, & Réale, 2009). The change in phenotype that results from interactions is a form of phenotypic plasticity (hereafter social plasticity).

Interacting phenotypes theory has used quantitative genetic models to show how evolutionary trajectories are altered by social plasticity (Bailey & Hoskins, 2014; Bailey & Zuk, 2012; Bijma, Muir, Ellen, Wolf, & Van Arendonk, 2007; Bijma, Muir, & Van Arendonk, 2007; Bijma & Wade, 2008; McGlothlin, Moore, Wolf, & Brodie, 2010; Moore, Brodie, & Wolf, 1997; Wolf, Brodie, & Moore, 1999). Indirect genetic effects, which occur when one individual's genes affect another individual's phenotype, may either amplify or decrease the amount of genetic variance available to selection. This process could quicken or slow the pace of evolutionary change and may also cause coevolution of otherwise uncorrelated traits (Moore et al., 1997). The effect of social plasticity on evolutionary processes, including those captured by quantitative genetic models, depends on the pattern of social interactions occurring within a population, that is, who interacts with whom and with what frequency or intensity. Early interacting phenotype models focused solely on simple dyadic interactions (Moore et al., 1997), and later attempts included unstructured interactions within larger groups (Agrawal, Brodie, & Wade, 2001; Bijma & Wade, 2008; Bijma, Muir, Ellen, et al., 2007; McGlothlin & Brodie, 2009; McGlothlin et al., 2010). However, none of these models have explored more realistically structured interactions where the strength of associations may vary across dyads and where individuals may not interact with every other member of their group. It is therefore unclear whether the conclusions from interacting phenotype models are generally applicable to most animal populations.

In nature, social interactions more often resemble structured networks than dyads or nonoverlapping groups. Social network analysis provides a powerful tool for quantifying the structure of such interactions (Farine & Whitehead, 2015; Whitehead, 2008) and its impacts on social processes (Aplin, Farine, et al., 2015; VanderWaal et al., 2016). Social network analysis uses information about who interacts with whom to link individual interactions to overall population‐level social structure (Hinde, 1976; Whitehead, 2008). In contrast to simpler models of social structure, social networks can capture variation in both the immediate social environment that individuals experience (i.e., who each individual interacts with directly) and the individuals’ positions within the overall social structure of the group (i.e., how central an individual is in stabilizing or favoring a particular social structure). Combining this greater realism when quantifying social structure, that is, the patterns of connections in a social network, with the ability to make formal predictions about phenotypic evolution has the potential to significantly expand our understanding of the evolution of social traits (Fisher & McAdam, 2017).

In this study, we investigate how social structures shape the impact of social plasticity on the amount of phenotypic variance available for selection and on the evolutionary response of traits to selection. First, we expand models of interacting phenotypes (McGlothlin et al., 2010; Moore et al., 1997; Wolf et al., 1999) to describe how varying aspects of social structure, such as strengths of connections between group members and preferential association based on phenotypic similarity, impact phenotypic variation, and evolution. Second, we create replicate groups of individuals with structured social interactions using agent‐based simulations to analyze how social structure influences distributions of phenotypes and the ability to respond to natural selection. We focus on the number of connections observed among group members (i.e., network density: the sum of all present edge weights divided by the possible sum of edge weights if the network were fully connected), and the degree to which individuals can bias the strength of their interactions with others that have a similar phenotype (i.e., network homophily). Although the parameters of our analytical model are not identical to those of our simulation model, they are analogous (i.e., mean connection strength is related to network density and phenotypic assortment is related to homophily), allowing us to compare the conclusions of the two approaches.

**Figure 1:**
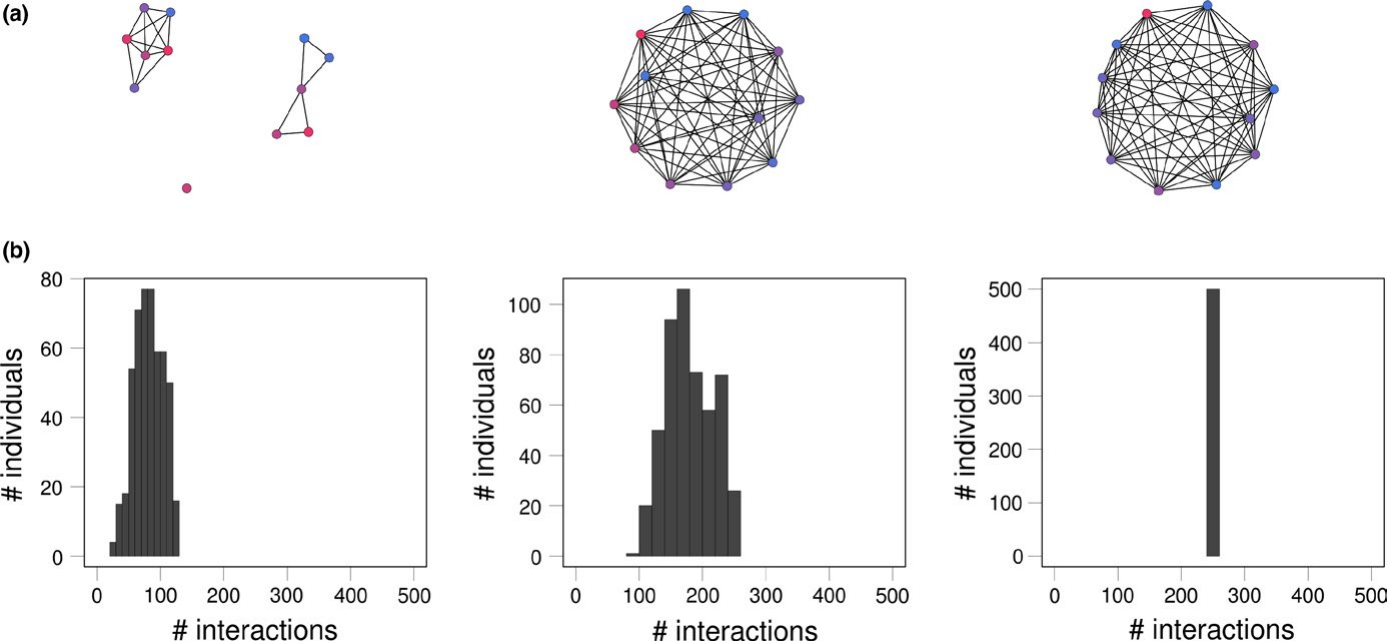
(a) Sample networks generated using three values of *r* (0.05, 0.15, and 0.9), creating networks of varying densities: low (left), intermediate (center), and high densities (right, *N*_group_ = 12). (b) For each level of density, individuals also varied in the number of interactions with other group members, shown at low (left), intermediate (center), and high (right) network densities (*N*_group_ = 500)

We predict that social plasticity should have a minimal effect on phenotypes and on their variation when connections among individuals are weak (at low network densities; Figure 1, left panels) because all individuals experience weaker effects of the same social environment (they are disconnected). Likewise, we predict there will be minimal variation in indirect genetic effects among individuals when connections are strong (at high network densities; Figure 1, right panels) because all individuals interact equally and with the same group (everyone excluding themselves). Thus, the social environment experienced by each individual should be very close to the average phenotype of the population. Networks with intermediate network densities (Figure 1, middle panel) have a greater scope to exhibit variation in local social structure resulting in variation in the social environment experienced by individuals. Next, we predict that homophily and social plasticity should interact in a similar way as do relatedness and indirect genetic effects in dyadic models (McGlothlin et al., 2010). Specifically, when social plasticity causes individuals to become more similar, adding preferential assortment should lead to an increase in phenotypic variation and an enhanced response to selection. Conversely, when individuals express heterophily (disassortative association by phenotype), we expect indirect genetic effects to decrease the ability of the trait to exhibit change in response to selection.

## 2 METHODS

### 2.1 An analytical model integrating connection strength and phenotypic assortment

We develop an analytical model of interacting phenotypes in a generalized social network. In our model, the average social plasticity of a group of individuals is represented by an interaction coefficient (ψ_*g*_), which measures the overall phenotypic effect of an individual's social partners’ phenotypes on its own phenotype. The phenotypic changes resulting from social plasticity are modulated by the overall mean strength of the connections among individuals (i.e., individuals are embedded within a weighted network with connection strengths ranging from 0 to 1, and the network density is the sum of all present edge weights divided by the possible sum of edge weights if the network were fully connected). While the product of social plasticity and connection strength could be modeled as a single parameter, we prefer to retain the distinction between connection strength and plasticity to keep our model compatible with empirical studies of indirect genetic effects (e.g., Cappa & Cantet, 2008) and allow extensions of this model in the future. We allow connection strengths to depend upon the nonplastic component of the phenotype (phenotypic assortment). Such assortment is analogous to homophily when assortment is positive (individuals seek similar partners) and heterophily when assortment is negative (individuals avoid similar partners). We followed the approach of Moore et al. (1997), ignoring the potential for direct social effects on fitness (social selection or group selection; Wolf et al., 1999). Although our model considers only a single trait for simplicity, it does not consider the possibility for feedback effects on phenotypes, making our model analogous to the two‐trait model with nonreciprocal effects of Moore et al. (1997). We will treat phenotypic feedback in a future contribution. The model is presented in its entirety in the Appendix, but we report its main results below (see Results).

### 2.2 Simulation overview

To investigate how social plasticity and social structure can affect the extent of phenotypic variation observed within populations and evolutionary change, we simulate groups of individuals with a fixed interaction coefficient (ψ_*g*_) but varying social structures (network density and homophily/heterophily). Each group has its own unique network of interactions. From these interactions, we calculate the phenotype that each individual in the group would express given social plasticity. We then calculate the phenotypic mean of the group, the variance in indirect genetic effects experienced among individual group members, the correlation between individuals’ genetic tendencies and the indirect genetic effects they experience, and the overall phenotypic variance of the group. We also calculate the overall genetic variance as the variance in total breeding values. Total breeding values are defined as the sum of individuals’ direct breeding values and their social breeding values (i.e., the effect of their genes on others via indirect genetic effects; see Appendix and McGlothlin & Brodie, 2009). Finally, we analyze how these four components vary as a function of network characteristics. The code used to generate these simulations is available on Dryad.

### 2.3 Generating social networks

We simulate replicated networks of varying density and homophily. Each replicate can be considered as a (small) population or as a distinct group embedded within a larger metapopulation. To generate networks of interaction, we first generate pairs of coordinates from a uniform distribution (range 0–1) for individuals. To allow each individual to interact more strongly with some of its conspecifics relative to others, we allow individuals to move toward their nearest neighbor by 15% of the distance between them. Varying this percentage does not influence our results (results not shown). To determine the connection strengths between each pair of individuals in our simulations (*s*), we calculate the Euclidean distance between them and use an exponential decay function generating a decreasing connection strength as a function of the distance between any two individuals. Thus, we assume that the connection strength between two individuals is $s=e^{-{\mathrm{distance}}^{2}/r}$, where *r* represents an interaction range. To avoid a fully connected network (where everyone interacts with everyone else), we remove very weak interactions (i.e., interactions with an edge weight of less than 0.05). Our approach allows us to generate networks of increasing densities by increasing the parameter *r* so that two individuals would be more strongly connected given a fixed distance between them. We increase *r* from 0 to 9 in 16 steps (at an increasing rate as the effects of *r* are not linear). Groups are generated with both 20 and 50 individuals, and we generate 50 replicate groups for each value of *r*.

Because the resulting network density (the sum of all present edge weights divided by the possible sum of edge weights if the network were fully connected) for a given value of *r* is stochastic, not deterministic, we report our results as a function of network density, which was calculated using the R package *assortnet* (Farine, 2014). Stated another way, network density is an emergent property of varying *r*, not a parameter of our simulations. Our approach is most immediately applicable to situations where individuals are actually distributed in two‐dimensional space and interact more or less intensely as a function of the distance (Farine, 2015). Such an interaction structure applies directly to situations such as competition among neighboring plants (e.g., Cappa & Cantet, 2008) or territoriality in animals (e.g., Royle, Hartley, Owens, & Parker, 1999) but is generalizable a wide variety of other more complex situations that may not involve a spatially explicit component.

Individuals can often choose to connect more strongly with some individuals than others. In many species, individuals preferentially interact with partners that are similar (i.e., under assortative mating, or during cooperative interactions) or dissimilar to them (i.e., under disasortative mating or because of division of labor and social heterosis, see Nonacs & Kapheim, 2007). Hence, networks can exhibit assortment (associations between individuals that are similar and/or avoidance of dissimilar individuals, Farine, 2014). First, we study the effect of randomly occurring network assortment in the simulated groups. To explicitly investigate the impact that interaction preferences can have on evolutionary processes, we then allow individuals to reduce the strength of their interaction with nonpreferred affiliates (e.g., with dissimilar individuals in the case of homophily, or with similar individuals in the case of heterophily). We thus modify the function used to calculate the strength of interaction (*s*) to $s=e^{-{\mathrm{distance}}^{2}/r}\times H\left(\frac{1}{1+{\text{exp}}^{-20|x-y|}}-0.5\right)+0.5$ which generates a sigmoidal function with a magnitude of *H* (the level of homophily ranging between 0 and 0.2) as a function of |*x* − *y*|, or the difference in the phenotypes of the individuals. If two individuals are identical, the strength of their interaction is multiplied by either ~0 or ~1 if modeling heterophily or homophily, respectively. Their connection strength is multiplied by 0.5 if their phenotypic difference (|*x* − *y*|) is average. As with network density, we report the measured network assortment calculated using the R package *assortnet* (Farine, 2014).

### 2.4 Generating individual phenotypes

We simulate individual phenotypes using the equation

(1) $$z=a+e+\;\frac{\psi_{g}}{n-1}\;\sum_{i=1}^{n-1}s_{i}\left({a_{i}}^{\prime }+{e_{i}}^{\prime }\right),$$

where the summation is taken over all possible *n* − 1 social interactions involving the focal individual (i.e., where s_*i*_ **> 0). This assumes no phenotypic feedback, but makes no further simplifying assumptions (see also Appendix). Individual breeding values (*a*) are sampled from a uniform distribution ranging from −1 to 1 (and thus with an average of 0). Nonsocial environmental effects (*e*) are also sampled from a normal distribution (mean = 0 and variance = 0.0625, a fifth of the average variance in breeding values). In absence of any social interaction, an individual's phenotype is predicted by direct genetic effects, and as a result, the population mean should be 0. When social interactions are present, an individual's phenotype also depends on the average breeding values and nonsocial environmental effects of its social partners ($a_{i}^{\prime }$ and $e_{i}^{\prime }$, respectively), which we weight by the strength of their social interactions *s*_*i*_. Individuals with no connection strength do not contribute to the phenotype as *s*_*i*_ **= 0. In our simulations, all individuals have the same interaction coefficient (ψ_*g*_). We also investigate whether our results depended on the distribution of breeding values by running additional simulations where individual breeding values are sampled from a normal distribution (mean = 0 and variance = 1). In the results, we point out where such a change in distribution affects our results. In previous models (McGlothlin & Brodie, 2009; McGlothlin et al., 2010), ψ_*g*_ has been constrained to lie between −1 and 1 for two reasons. First, values of ψ_*g*_ greater than 1 can lead to unreasonable phenotypic values (particularly in models that include phenotypic feedback). Second, phenotypic values are often standardized to a mean of zero and unit variance for analysis, which should result in ψ_*g*_ values between −1 and 1. In our simulations, the mean and variance of individual phenotypes varied in each group because of sampling, which made such standardization difficult. We chose to use unstandardized trait values and a large ψ_*g*_ value (4) in all simulations. Such a value of ψ_*g*_ which would yield an average standardized value of ψ_*g*_ of ~0.30, which is comparable to empirical ψ_*g*_ values reported in the literature (e.g., Bailey & Hoskins, 2014; Bailey & Zuk, 2012). Using this large value facilitates visualizing social effects and does not lead to unreasonable phenotypic values due to the absence of phenotypic feedback in our model. As noted in the Appendix, ψ_*g*_ is multiplied by average connection strength (network density) when calculating phenotypes, which will reduce the effect of ψ_*g*_ except in fully connected networks. As predicted by the analytical model, increasing the strength of social plasticity (i.e., how much an individual changed his phenotype in response to his social partners, the absolute value of ψ_*g*_) amplifies all the patterns we report below (see Results section). However, because such increases are intuitive and of lesser interest, we do not report the results of analyses varying ψ_*g*_. Using the code available as Figure S1, the reader can generate figures that are comparable to the ones we present below for any value of ψ_*g*_.

## 3 RESULTS

### 3.1 Analytical results

In a generalized social network, the predicted phenotypic mean is

(2) $$\overset{=}{z}=\left(1+\;\psi_{g}\overset{=}{s}\right)\overset{=}{a},$$

where ψ_*g*_ represents the strength of social plasticity, $\overset{=}{s}$ represents the average connection strength within the network across all replicate groups (i.e., network density), and $\overset{=}{a}$ is the average individual genetic value. The predicted phenotypic variance within a group is

(3) $$\mathrm{Var}\left[z\right]=G+E+\psi_{g}^{2}\mathrm{Var}\left[\bar{sa^{\prime }}+\bar{se^{\prime }}\right]+2\psi_{g}\mathrm{Cov}\;\left[a\;+e,\bar{sa^{\prime }}+\bar{se^{\prime }}\right],$$

where *G* indicates additive genetic variance and *E* indicates environmental variance. The third term above represents the among‐individual variance due to social interactions. This term should increase somewhat with homophily, but should depend most heavily on network density. The variance in social environment experienced by individuals should be at a maximum at intermediate $\overset{=}{s}$ and should decrease at very high values of $\overset{=}{s}$ as social interactions become more homogenous (i.e., everyone interacts with everyone). The fourth term will be most influenced by homophily (or heterophily) because associating with similar (or different) individuals will cause the covariance to increase (or decrease). The multiplication by ψ_*g*_ will cause phenotypic variance within a group to increase with homophily under positive values of ψ_*g*_ and decrease with homophily when ψ_*g*_ is negative. This term should also be influenced by average connection strength ($\overset{=}{s}$) in the absence of homophily, becoming negative at high connection strengths because individuals are not included as part of their own social environment.

Response to selection is predicted by the equation

(4) $$\Delta \overset{=}{z}\approx \left(1+\psi_{g}\overset{=}{s}\right)G\left(1+R\psi_{g}\right)\beta ,$$

where *R* is a general measure of the strength of homophily (see Equation A19 in Appendix) and β is the selection gradient (see Appendix). This equation shows that the amount of genetic variance available for response to selection at the population level should depend on (1) the degree of social plasticity, (2) the average connection strength (which should increase with mean connection strength), and (3) the amount of association between individuals that is based on genetic value similarity (homophily or heterophily). This model is nearly identical to previous models with simpler group structure (McGlothlin et al., 2010) except for the inclusion of the connection strength ($\overset{=}{s}$) and the replacement of relatedness with homophily/heterophily (*R*). These analytical results provide predictions that we test below using individual‐based simulations.

### 3.2 Network density

**Figure 2:**
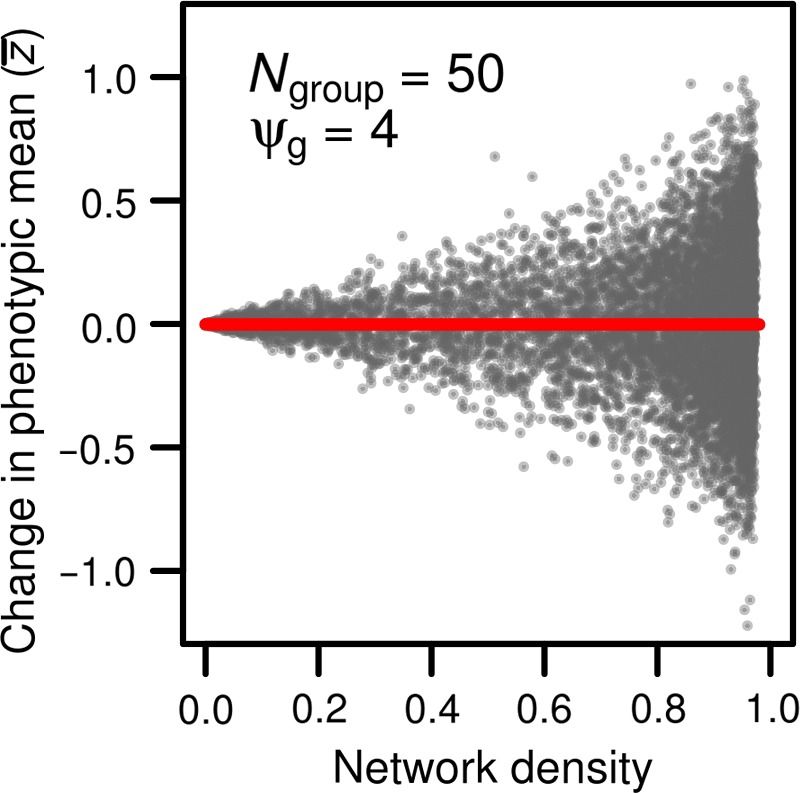
The change in average phenotype of individuals in a network as a function of the network density (red line). The change in average phenotype is expressed relative to the phenotype expected in absence of interactions among individuals (i.e., 0). Each dot represents a simulated network, or group of 50 individuals (*N*_group_ = 50). Indirect genetic effects were generated using a ψ_g_ of 4. As density increased, we observed a greater variation in mean phenotype among groups (gray dots)

In our simulations, increasing network density, which is analogous to increasing mean connection strength in the analytical model, does not lead to an increase in phenotypic mean on average (i.e., across all groups, Figure 2, red line). However, at higher network densities, there is much greater variation among groups in their phenotypic mean ($\text{Var}[\overset{=}{z}]$, Figure 2, gray dots). This occurs because although the mean genetic value across all simulations is zero, this value can differ across groups due to sampling. As predicted by Equation 2, increasing network density (or $\overset{=}{s}$) increases the importance of the group genotypic composition in determining the effects of indirect genetic effects, magnifying differences among groups in genetic value ($\overset{=}{a}$) across replicate simulation runs. This amplification effect is also observed when social plasticity (ψ_*g*_) is negative (see Figure S2 upper panel) and is stronger when breeding values follows a uniform distribution (i.e., when there are more individuals with extreme breeding values, Figure 2) than when they follow a Gaussian distribution (i.e., when there are fewer individuals with extreme breeding values, see Figure S3). The amplification effect is also more pronounced in smaller groups (see Figure S4).

**Figure 3:**
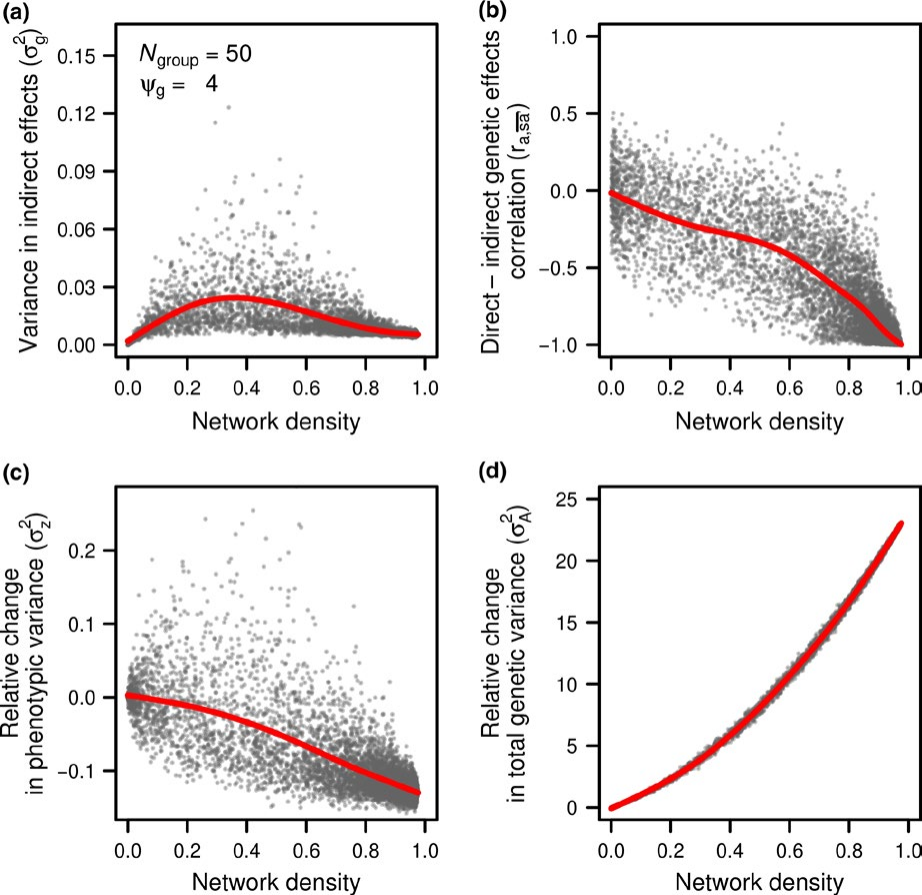
Effects of network density on (a) the variance in indirect effects experienced by individuals, (b) the correlation between direct and indirect genetic effects experienced by individuals, (c) the change in phenotypic variance within groups relative to the genetic variance (i.e., the phenotypic variance in absence of interactions), and (d) the change in total genetic variation relative to the genetic variance (i.e., variance of individual direct genetic effects and indirect genetic effects imposed to others). Each dot represents a simulated network, or group of 50 individuals (*N*_group_ = 50). Indirect genetic effects were generated using a ψ_g_ of 4

The phenotypic variance in indirect effects within each group is maximal at intermediate network densities (Figure 3a). When individuals have few connections (and connection strength is weaker), the scope for indirect effects to differ among members of a given group is narrow, thereby decreasing the contribution of social interactions to phenotypic variance (Figure 3a). Likewise, in groups where individuals are highly connected (high network density), the social environment experienced by each individual is closer to the average breeding and environmental value of the population (which is 0 in our simulations). In other words, Var[*a*′], and consequently $\text{Var}[\bar{sa^{\prime }}]$, within groups is small at high densities (see Equation 3). However, at intermediate densities, indirect effects had the potential to make a large contribution to phenotypic variance, although this effect was highly variable across simulations.

In the absence of homophily/heterophily, high network density leads to a negative correlation between direct and indirect genetic effects (Figure 3b). At low network densities, this correlation is expected to be zero because individuals associate at random (although this would not be the case if there was any spatial assortment by phenotype). However, at high densities, this correlation becomes negative even though individual association is also random. This effect arises because individuals are not counted as part of their own social environment, and as network densities increase, the importance of this difference becomes magnified. At the highest network densities, social environments are indistinguishable except for this effect of excluding oneself, thus leading to a direct–indirect correlation of −1. Smaller groups will exhibit this pattern to a stronger extent than larger groups (see Figure S5, also McDonald, Farine, Foster, & Biernaskie, 2017). The combined impact of the effects of network density on the variance in indirect genetic effects and on the covariance between direct and indirect genetic effects is a relative decrease in phenotypic variation within groups (compared to the phenotypic variation in absence of any interactions) as network density increases (Figure 3c). Opposite patterns are observed when ψ_*g*_ is negative (Figure S6). Finally, we note that although the mean phenotypic variation within each group decreases with increasing network density, we find that the variation among groups (each dot in each panel of Figure 3c represents one group) is maximized at intermediate network densities.

**Figure 4:**
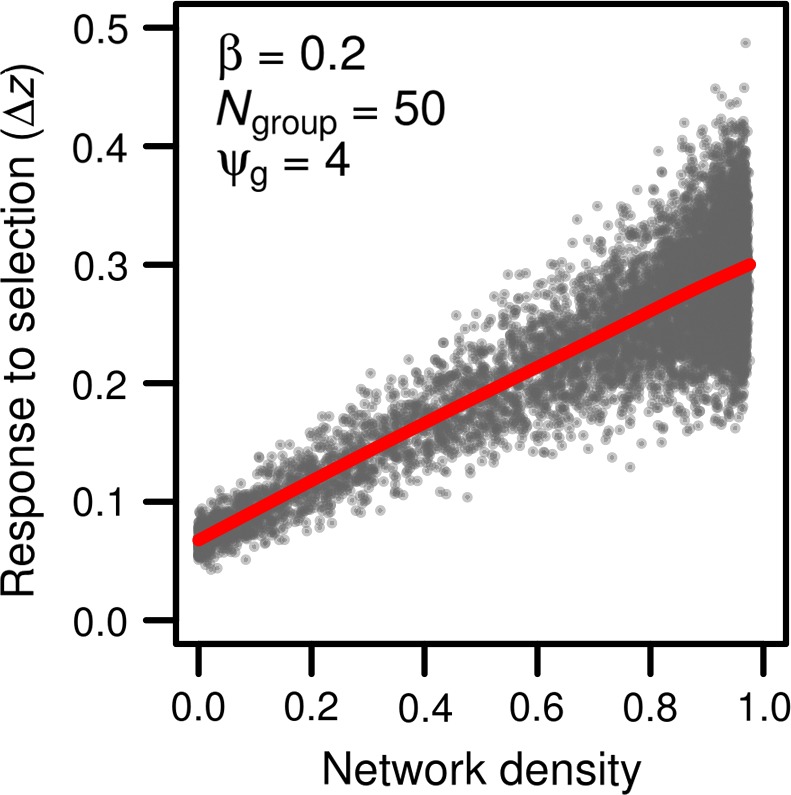
Evolutionary response of phenotype to a selection gradient varies with network density. The average change in phenotype mean resulting from selection increased with network density (red line). Groups with denser networks of interactions exhibited more variation in the change in phenotypic mean (gray dots). Each dot represents a simulated network, or group of 50 individuals (*N*_group_ = 50). Indirect genetic effects were generated using a ψ_g_ of 4

Although the phenotypic variance typically decreases with increased network density, the variance in total breeding values (relative to the genetic variance in absence of any social interaction) has the greatest increase at highest network densities (Figure 3d, red line). This is because the expected variance in total breeding values is equal to ${\left(1+\psi_{g}\overset{=}{s}\right)}^{2}G$ (see Equation A12). That is, the variance in total breeding values does not depend on the variance in indirect genetic effects, nor on the covariance between direct and indirect genetic effects, which lead to the decrease in variance shown in Figure 3c. Rather, the variance in total breeding values is a function of direct genetic effects and effects of an individual's genes on others, the latter of which becomes inflated at higher densities. The response to selection depends on the covariance between total breeding values and phenotypic values, which is expected to increase linearly with density (Equation A15). When we subject groups to a selection gradient of 0.2, networks with increasing densities exhibited an increased evolutionary response to selection (Figure 4, red line). Increasing network density also increases the variance in response to selection among groups (Figure 4, gray dots). This likely happened because individuals adjust their phenotype to the average social environment that they experience. Thus, at higher network densities, individuals with extreme phenotypic values have a disproportionate impact on the average social environment experienced by individuals. Small differences in the phenotypic values of extreme individuals from group to group create differences in the covariance between the phenotypic variance and the total breeding values among groups and increasing the variance in response to selection among groups. In agreement with this, extreme individuals also generate greater phenotypic variance among groups at higher network densities (Figure 2) and when individual phenotypes follow a uniform distribution (more individuals with extreme phenotypes) than a normal distribution (fewer individuals with extreme phenotypes, See Figure S3).

### 3.3 Homophily

**Figure 5:**
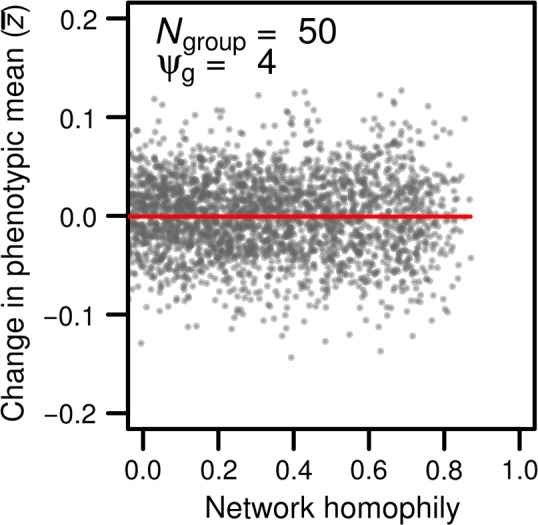
The average phenotype of individuals as a function of network homophily (red line). Each dot represents a simulated network, or group of 50 individuals (*N*_group_ = 50). Indirect genetic effects were generated using a ψ_g_ of 4. Networks could not reach homophily values of 1 because we used a continuous trait value rather than discrete values (see Farine, 2014)

**Figure 6:**
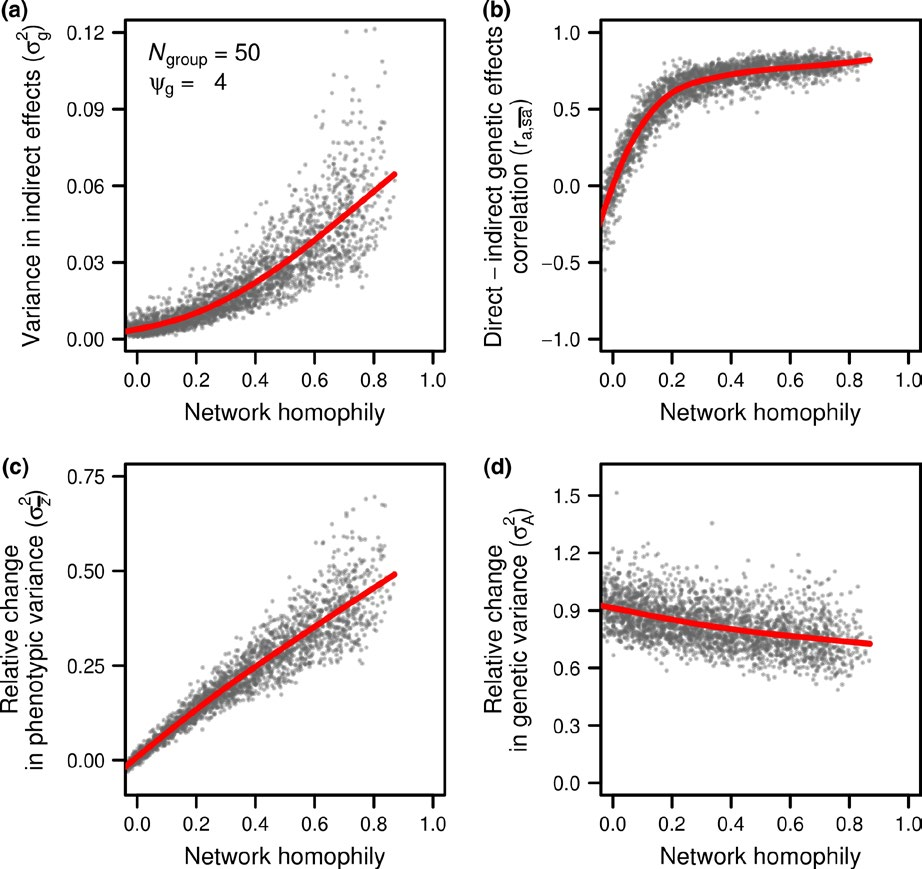
Effect of network homophily on (a) the variation in indirect effects, (b) the correlation between direct and indirect genetic effects, (c) the relative change in phenotypic variance within groups, and (d) the relative change in total genetic variation. Each dot represents a simulated network, or group of 50 individuals (*N*_group_ = 50). Indirect genetic effects were generated using a ψ_g_ of 4. Homophily simultaneously increases variation among individuals in indirect genetic effects within groups, and generates a strong positive covariance between direct and indirect genetic effects, thereby substantially increasing the phenotypic variance observed within groups. However, this increase in phenotypic variance is not associated with any change at the genetic level

Allowing individuals to increase the strength of their connections with conspecifics that have similar phenotypes (i.e., increasing homophily) has no effect on the mean phenotype of each group (Figure 5), which is consistent with our analytical model (Equation 2). However, increased network assortment is associated with an increase in the variance in indirect genetic effects experienced by individuals in a given group (see the third term in Equation 3, Figure 6a). Because individuals interact more strongly with conspecifics that have similar breeding values (high with high, low with low), the direct and indirect genetic contributions to phenotypes act in concert and covary positively (fourth term in Equation 3, Figure 6b). These two effects contribute toward increasing phenotypic variance observed within a given group (Figure 6c). These effects are reversed when ψ_*g*_ is negative: direct and indirect contributions to individual phenotypes act to oppose each other, thereby reducing the amount of phenotypic variance observed in the population (see Figure S7). Increasing network assortment also leads to a small decrease in the variance in total breeding values (Figure 6d, red line). This is attributable to the slight decrease in density associated with higher levels of network assortment (i.e., individuals have the ability to reduce the connection strength with particular group members), a phenomenon not captured by our analytical model.

**Figure 7:**
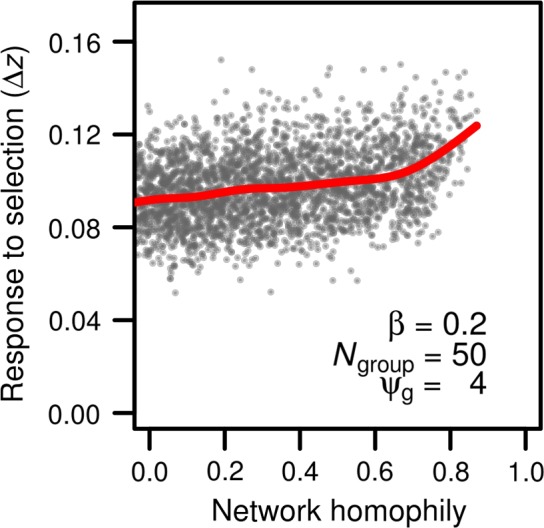
Evolutionary response of phenotype to a selection gradient increases with network homophily (red line and gray dots). Each dot represents the change in mean genotype across generations in a single simulated network, or group of 50 individuals (*N*_group_ = 50). The red line represents the average evolutionary response as a function of network density. Indirect genetic effects were generated using a ψ_g_ of 4

Applying selection to groups with varying network assortment shows that a synergy between direct and indirect genetic effects leads to an increase in evolutionary change with increasing network assortment (Figure 7, red line). This result is in accord with the predictions of our analytical model, which predicts an increased response to selection with increasing *R* (Equation 4). Although Equation 4 suggests density and homophily should have symmetrical effects, the increase seen in Figure 7 is not as dramatic as that in Figure 4, perhaps because of the concomitant decrease in density caused by allowing homophily in our simulations. Unlike network density, network assortment does not affect the variation of evolutionary responses among groups (Figure 7, gray dots).

## 4 DISCUSSION

In this study, we explore the consequences of social plasticity and the structure of interactions in shaping the amount of phenotypic variation within groups of interacting individuals and the evolutionary response of these groups to selection. We consider replicate groups with varying structures of interactions. Such replicates could be seen as multiple groups of individuals within a given population (e.g., tribes, packs, or colonies), or as multiple isolated populations within an ecological community. Through an analytical model and agent‐based simulations, we show that the structure of social networks modulated the impact of indirect genetic effects on the amount of phenotypic variance available for selection. Our results emphasize that the number and strength of connections among individuals (network density) as well as preferential associations among individuals with a similar phenotype (network assortment) have important effects on the contribution of indirect genetic effects. Network density and assortment also modulate the ability for traits to exhibit evolutionary change in response to selection. Increasing the number of interactions among group members (network density) increases the average evolutionary response of groups to selection and increases the variation in response to selection among groups. By contrast, increased network assortment leads to an increase in the average evolutionary response of groups to selection, but does not affect the variation in evolutionary response among groups. Our results have widespread implications for studies of social evolution, multilevel selection, and the emergence of keystone individuals (Modlmeier, Keiser, Watters, Sih, & Pruitt, 2014) and niche‐constructing traits (e.g., Sih & Watters, 2005).

### 4.1 Comparison with earlier interacting phenotype models

Our analytical results show that, given some simplifying assumptions (most importantly, ignoring the potential for phenotypic feedback), considering more general social structures does not greatly alter the conclusions of earlier models of interacting phenotype evolution (e.g., McGlothlin et al., 2010; Moore et al., 1997). First, indirect genetic effects still alter the response to selection by increasing the amount of genetic variance exposed to selection. Note that because we do not model feedback, this effect is directional, with positive ψ_*g*_ (i.e., becoming more similar to one's neighbors) increasing the response to selection and negative values (i.e., becoming more different from one's neighbors) decreasing it. Second, homophily/heterophily plays a role similar to relatedness in previous models in altering the response to selection (McGlothlin et al., 2010). Indeed, McGlothlin et al. (2010) noted (as have many others) that relatedness per se is just a special case of phenotypic assortment.

The most notable difference between our model and earlier models is that individuals are allowed to differ in their influence on social plasticity due to variation in connection strength (*s*). Perhaps unsurprisingly, response to selection is more strongly influenced by social interactions in denser networks where interactions are stronger and more common, an effect that is apparent in both our analytical and simulation results. In our simulation results, we also find that variation in response to selection among increases with greater connection strength. This could occur because in some groups, the most well‐connected individuals happen to have relatively high or low breeding values. These well‐connected individuals would then have a disproportionate effect on the phenotype of others (relative to individuals closer to the population's average breeding value) by exerting a strong effect on the mean social environment experienced by individuals. In doing so, well‐connected extreme individuals can generate variability in the covariance between breeding values and phenotype, and thus in the response to selection. Future models should allow connection strength to display a heritable component, which would allow the social network structure itself to evolve in response to such effects. Studies in the wild have shown that position in a social network may be repeatable and have fitness consequences, suggesting that connection strength may be an evolvable trait (Aplin, Firth, et al., 2015; Formica, Wood, Cook, & Brodie, 2017; Formica et al., 2012).

### 4.2 Groups with denser connections exert a stronger “pull to the mean”

Our simulations show that groups where individuals have an intermediate number or strength of social connections (i.e., groups with intermediate network densities) allow for the greatest within‐group variation in social environments experienced among individuals. This pattern arises within a generation due to social plasticity, rather than as a response to any selective pressure. By generating a greater range of phenotypes and social environments, groups with an intermediate density of social interactions could provide the basis for the emergence and evolution of alternative social phenotypes (Bergmüller & Taborsky, 2010; Sinervo & Calsbeek, 2010). When social phenotypes are subject to indirect genetic effects, the maintenance of variation in social behavior should thus be observed more frequently in groups with intermediate connectedness.

While low to intermediate densities of social interactions offer the widest scope for promoting individual variation in phenotype, groups with high densities exhibit a very different pattern. As the number and strengths of interactions increases among group members, indirect genetic effects experienced by each individual become more similar, because all group members start to become connected to one another, and these connections are uniformly strong. In addition, as networks become more connected, individuals at the extremes of the distribution of genetic tendencies in the groups (i.e., the individuals furthest from the average breeding value) tend to experience a social environment exerting an opposing effect on their phenotype (i.e., $\text{Cov}[a+e,\bar{sa^{\prime }}+\bar{se^{\prime }}]$ in Equation 3 becomes more negative). This “pull to the mean” is stronger in smaller groups than in larger ones (see Figure S5), and decreases the population phenotypic variance when ψ_*g*_ is positive (e.g., when individuals increase their aggressiveness in response to the aggression they receive) and increases it when ψ_*g*_ is negative (e.g., when individuals inhibit their aggressiveness in response to the aggression they receive). When individuals exhibit important phenotypic plasticity, such an effect appears similar to social conformity, where individuals express more similar phenotypes as groups than in isolation (e.g., Herbert‐Read et al., 2013; Magnhagen & Bunnefeld, 2009). Conformity is usually thought to emerge when dissimilar individuals are at a fitness disadvantage. For example, predators tend to pick individuals that stand out among groups of prey, selecting for homogeneous groups (Landeau & Terborgh, 1986). We show here that conformist‐like patterns in behavior can emerge from very simple forms of social plasticity. Importantly, we show that behavioral conformity can emerge even when individuals are not actively copying the most abundant phenotype of their group, in absence of any selective pressure. Examples of highly connected groups in nature include choruses of males in some species of frogs producing mating calls or leks of males displaying to attract females and adjust their signaling effort in response to the signaling of their rivals (Boatright‐Horowitz, Horowitz, & Simmons, 2000; Simmons, Simmons, & Bates, 2008). When males are close to each other and each male can adjust its signaling intensity to that of all the other males, we expect this “pull to the mean” to decrease the variation in male signals (although social plasticity can take more complex patterns then is modeled here, Greenfield & Rand, 2000). This can decrease the effectiveness of female mate choice, and the intensity of sexual selection.

### 4.3 Groups with denser connections potentiated the effect of keystone individuals

An insight from our simulations is that whenever individuals adjust their phenotype to the average social environment that they experience, individuals at the extremes of the genotypic or phenotypic distribution have the greatest impact on the phenotype of other group members. This phenomenon arises because these individuals have the strongest impact on the mean social environment experienced by their conspecifics. The evolution of niche‐constructing traits, allowing some individuals to manipulate their group in a way that favors their own success (Saltz, Geiger, Anderson, Johnson, & Marren, 2016) or to have a disproportionate effect on their group (Keiser & Pruitt, 2014; Modlmeier et al., 2014; Pruitt & Pinter‐Wollman, 2015) has been the focus of much interest recently. Surprisingly, no study has explored the implications of the exact patterns of social plasticity for the identity and impact of such “keystone individuals” (Modlmeier et al., 2014). It is reasonable to assume that most animals exhibiting social plasticity should adjust their phenotype in response to the *average* social conditions that they experience (although in some cases, some individuals can adjust their phenotype to the maximum, or to the minimum phenotypic value of the individuals with whom they interact, see Dyer, Croft, Morrell, & Krause, 2009 for a potential example). Thus, our simulations suggest that the emergence of keystone individuals might be a phenomenon far more common than previously envisioned. Keystone individuals could emerge in any group of interacting individuals exhibiting phenotypic plasticity and dense interaction networks (as in the lekking example above). Hence the emergence of keystone individuals might not require a complex social system, division of labor (Pruitt, Bolnick, Sih, DiRienzo, & Pinter‐Wollman, 2016), collective behavior (Pruitt & Pinter‐Wollman, 2015), or niche‐constructing traits (e.g., aggressiveness, policing, or else, Chang & Sih, 2013), but should be most common in species where individuals respond to the behaviors of the majority of group members.

Denser interactions among individuals within a group potentiate the impact of individuals with extreme phenotypic values by allowing these to interact and affect the majority of their group members. Hence, the consequences of individual social plasticity tend to become much greater in groups as network density increases. This “amplification” effect is also stronger when genotypes follow a uniform distribution (i.e., when extreme genotypes are more frequent within groups) than when they follow a Gaussian distribution (i.e., when extreme genotypes are rare within groups). Network density increases the evolutionary response of groups on average, groups with denser connections also exhibit increased variation in evolutionary response to a given selection gradient. Such an increase in the variation in evolutionary response observed in our study is apparent because our approach allows us to relax the assumption that individuals within groups interact equally with all other group members by introducing a new parameter (average connection strength, $\overset{=}{s}$) into our model, and instead to explicitly explore the effect of connection density.

### 4.4 Homophily affects the amount of phenotypic variation within groups

Our simulations also investigate the consequences of social structure when individuals can interact preferentially with individuals that have a similar (i.e., homophily) or dissimilar (i.e., heterophily) phenotype. Network assortment (the network measure of homophily) does not have any impact on groups’ mean phenotype, but can generate a greater variation in social environments within groups. Hence, assuming that different immediate social environments can favor different (alternative) phenotypes within groups, our simulations suggest that homophily should be associated with the emergence of phenotypic variation among individuals (or social specialization, Bergmüller & Taborsky, 2010; Montiglio, Ferrari, & Réale, 2013). Homophily also has direct consequences for the evolution of alternative phenotypes by creating synergy or opposition between direct and indirect genetic effects. When social plasticity (ψ_*g*_) is positive (e.g., when interacting with an aggressive individual increases a focal individual's aggression) and individuals show a tendency to interact with similar phenotypes, the social environment acts to shape their phenotype in the same direction as their genotype. As a result, social interactions and social plasticity increase the phenotypic variance of the trait relative to a situation in which connections are random in terms of their traits. It also tends to increase the strength of the evolutionary response to selection in response to a given selection gradient. This increase in evolutionary response arises from the synergy between direct and indirect effect (or between social plasticity and homophily) rather than from a change in total genetic variation within groups.

### 4.5 Possible applications and tests of the model and future directions

Our model has implications for a wide array of study systems. Reaction norms and social network analysis are often used to investigate the expression of labile sexual traits during mating and social interactions. In many species of crickets, birds, or anurans, males sing to defend territories or attract mates. Individuals adjust the intensity of their calls in response to the calls made by neighboring males. In such systems the structure of interactions among males, which can depend on the spatial distribution of their territories, will potentially affect the phenotypic variation among males and eventually affect the sexual selection differential and mate choice by females. The strength of connections between males of known phenotype could be increased artificially using playbacks to simulate more frequent interactions among individuals (Dabelsteen & Pedersen, 1990; Otter et al., 2001) to monitor how such increases in connection strength affect the extent of phenotypic and genetic additive variation. Alternatively, one can manipulate the distribution of territories available and the patterns of interactions by controlling the location of nest boxes, food patches, or refuges. Such approaches could be particularly suitable for cavity‐nesting birds (Both, 1998).

Our model also studied the consequences of homophily for the evolutionary responses of phenotypic traits. Assuming individuals have some level of control on their interactions, homophily can be observed when individuals exhibit preferences to interact with conspecifics that are (dis)similar to them. Such preferences could explain the maintenance of altruistic behavior because of their potential role in shaping the selection pressures acting on altruism. Some work on fruit flies have reported that individual preferences for particular social environments is associated with genetic variation and can thus potentially evolve (e.g., Saltz & Foley, 2011). One could test our model in such study systems by either allowing or preventing the expression of social preferences (through mixing of individuals or manipulating their interactions). Alternatively, homophily can also be observed when individuals segregate in the environment, as a function of their phenotype (Helfenstein, Danchin, & Wagner, 2004; Ward, 1993; Ward & Porter, 1993). For example, more and less aggressive individuals segregate in patches with different densities of conspecifics (Duckworth, 2006). Thus, by manipulating the heterogeneity and the scale at which it is observed (i.e., the size of patches of different habitat), one could test the predictions of our model. Future work will expand the model we presented here to analyze the consequences of such traits for the maintenance of phenotypic variation and the evolution of social structure.

One limitation of our model is that we assume both phenotypic feedback (Moore et al., 1997) and social selection (Wolf et al., 1999) to be absent. Including feedback may alter the conclusions we have presented here, as feedback effects may cause social plasticity to move through networks in counterintuitive ways. It is possible that network metrics beyond connection strength (such as clustering coefficients, betweenness, etc.) may influence whether feedback contributes significantly to variation and response to selection. Regarding social selection, we have shown here that connection strength contributes to among‐group variance, which should intensify response to higher levels of selection such as social selection. We will treat both feedback and social selection in future contributions.

## 5 CONCLUSION

We present both an analytic model and simulations extending previous work on social phenotypic plasticity to more general social structures. Our study generates a first set of very general and testable predictions on the role of social structure in modulating the consequences of social plasticity. We show that the basic characteristics of the structure of interactions among individuals in groups and populations can have impacts on the consequences of social plasticity for the mean phenotype expressed by the individuals, the extent of phenotypic variation available for selection, and for the ability of the population to respond to selective pressures. We hope that future studies will be able to test our predictions empirically by applying our approach to the study of social behavior (e.g., aggression and cooperation), social information use, or alternative mating tactics in populations with varying patterns of social or mating interactions. From a theoretical perspective, future research would warrant investigating the link between individual position in social networks and the indirect genetic effects it experiences, and thus whether factors other than individuals’ breeding values can lead to keystone individuals. Further, in our study, we assume that all individuals expressed plasticity identically. However, individuals have also been shown to vary in their plasticity (Dingemanse, Kazem, Réale, & Wright, 2010 Nussey, Wilson, & Brommer, 2007; Westneat, Hatch, Wetzel, & Ensminger, 2011) and such variation may alter the predictions of interacting phenotype models (Kazancıoğlu, Klug, & Alonzo, 2012). Linking individual differences in both social position and plasticity could yield new insights and a greater understanding of the evolutionary mechanisms that underpin phenotypic variation within individuals, between individuals, and among populations.

## CONFLICT OF INTEREST

None declared.

## AUTHOR CONTRIBUTIONS

POM and DRF built the simulations, and JWM developed the model. POM drafted the manuscript and produced the figures. All authors contributed to revisions and approved the final version of the article.

## APPENDIX 1

### 1.1

#### PHENOTYPIC MEAN AND VARIANCE

Following Moore et al. (1997), we model a single phenotype of an individual (*z*) as an additive combination of the effects of its own genes (i.e., direct genetic effects, *a*), the nonsocial environment it experiences (*e*), and a plastic component that is some function of the phenotypes in its social group. Moore et al. (1997) originally modeled the social effect as a function of the phenotype of one interacting individual. McGlothlin and Brodie (2009) and McGlothlin et al. (2010) considered social effects in a population consisting of groups of *n* individuals, where interactions were assumed to occur simultaneously within a group, with all individuals within a group having the same strength of interaction. Here, we consider a more general social structure, where individuals interact with all possible social interactants in group size *n* with variable connection strength *s*_*i*_, ranging from 0 to 1, giving

(A1) $$z=a+e+\psi \sum_{i=1}^{n-1}s_{i}z_{i}^{\prime },$$

where the summation is taken across all *n *− 1 possible social interactions for each individuals and ψ represents the interaction coefficient, which determines the strength and direction of the indirect phenotypic effect of individual social interactants. Here and elsewhere, a prime indicates a parameter belonging to a social partner. Any social network structure can be modeled by changing the distribution of *s*. High‐density social networks with strong connections will have many large values of *s*, while sparser networks with weaker connections will have many weak *s* values, with many equal to zero.

Considering phenotypic feedback (as do Moore et al., 1997) in such a model is complex, because interactions may vary across individuals. Making the simplifying assumption that phenotypic feedback can be ignored, we can write the social group effect as a function of genetic and environmental components:

(A2) $$z=a+e+\psi \sum_{i=1}^{n-1}s_{i}\left(a_{i}^{\prime }+e_{i}^{\prime }\right).$$

This may also be written as

(A3) $$z=a+e+\left(n-1\right)\psi \left(\bar{sa^{\prime }}+\bar{se^{\prime }}\right),$$

where a single overbar indicates a mean taken across an individual's social group, that is, all other individuals in the group weighted by individual connection strengths (*s*). This can be simplified further by defining a group interaction coefficient such that

(A4) $$z=a+e+\psi_{g}\left(\bar{sa^{\prime }}+\bar{se^{\prime }}\right)$$

(cf. McGlothlin & Brodie, 2009; McGlothlin et al., 2010). Because many social interactions in a group are likely to be weak unless group density is quite high, ψ_*g*_ may often take large values. Taking the variance of this equation and assuming Cov[*a*,*e*] = 0 leads to Equation 3 in the text. Using the definition of covariance, this can be rewritten as

(A5) $$z=a+e+\psi_{g}\left(\overset{¯}{s}{\overset{¯}{a}}^{\prime }+\text{Cov}\left[s,a^{\prime }\right]+\overset{¯}{s}{\overset{¯}{e}}^{\prime }+\text{Cov}\left[s,e^{\prime }\right]\right).$$

The value of the two covariance terms represents how strongly strength of interaction depends on the genes and environment of individual social partners. This covariance is determined for each individual and may vary across individuals. These should be positive under homophily and negative under heterophily. Taking the mean and assuming $\overset{=}{e}=0$,

(A6) $$\overset{=}{z}=\overset{=}{a}+\psi_{g}\left(\bar{\overset{¯}{s}{\overset{¯}{a}}^{\prime }}+\bar{\overset{¯}{s}{\overset{¯}{e}}^{\prime }}+\bar{\text{Cov}[s,a^{\prime }]}+\bar{\text{Cov}[s,e^{\prime }]}\right),$$

where double overbars indicate global means and large overbars within the parentheses indicate means taken across individuals based on summary statistics of their social groups. This can be expanded as above to

(A7) $$\overset{=}{z}=\overset{=}{a}+\psi_{g}\left(\overset{=}{a}\overset{=}{s}+\text{Cov}\left[\overset{¯}{s},{\overset{¯}{a}}^{\prime }\right]+\text{Cov}\left[\overset{¯}{s},{\overset{¯}{e}}^{\prime }\right]+\bar{\text{Cov}[s,a^{\prime }]}+\bar{\text{Cov}[s,e^{\prime }]}\right).$$

The two new covariance terms represent the covariance between average connection strength of an individual and the phenotypes of other individuals in the social group. If the group size is large enough, these terms will go to zero because variance in ${\overset{¯}{a}}^{\prime }$ and ${\overset{¯}{e}}^{\prime }$ will approach zero. This leaves

(A8) $$\overset{=}{z}=\overset{=}{a}+\psi_{g}\left(\overset{=}{a}\overset{=}{s}+\bar{\text{Cov}[s,a^{\prime }]}+\bar{\text{Cov}[s,e^{\prime }]}\right).$$

The remaining covariance terms represent the average degree to which individual connection strengths are adjusted based on the phenotype of potential interactants. In the case of homophily or heterophily, individuals will make this adjustment based on their own phenotype, with large individuals having a positive covariance and small individuals having a negative covariance (or vice versa). Therefore, we expect these average covariance terms to tend toward zero. Making this assumption and rearranging provides Equation 2 in the text.

#### EXTENSION TO MULTIPLE GROUPS

Equation A8 is written assuming that the population consists of a single social network where individuals may interact in any way. Now assume that the population is divided into multiple groups where individuals may interact with any strength *s* within a group and do not interact at all between groups. Assuming the covariance terms in Equation A8 are zero and that environmental deviations do not vary among groups, the mean of each group will be

(A9) $$\overset{=}{z}=\left(1+\psi_{g}\overset{¯}{s}\right)\overset{¯}{a},$$

where overbars now indicate within‐group means. Assuming equal group size, the population mean is now

(A10) $$\overset{=}{z}=\overset{=}{a}+\psi_{g}\left(\overset{=}{a}\overset{=}{s}+\text{Cov}\left[\overset{¯}{s},\overset{¯}{a}\right]\right).$$

(If groups vary in size, then Equation A10 can be modified to give a weighted mean.) If connection strength has no heritable basis, then the covariance term become zero and Equation A10 collapses to Equation 2 in the text. We may calculate among‐group variance most easily by making a few assumptions that are also made in our simulations. First, as above, we assume that groups are of equal size. Second, we assume that mean interaction does not vary across groups, and $\overset{¯}{s}=\overset{=}{s}$. Making these assumptions,

(A11) $$\text{Var}\left[\overset{¯}{z}\right]={\left(1+\psi_{g}\overset{=}{s}\right)}^{2}\text{Var}\left[\overset{¯}{a}\right].$$

This shows that indirect genetic effects will magnify slight differences in genetic values across groups regardless of sign. Stronger mean connection strengths will intensify this effect.

#### RESPONSE TO SELECTION

Following Moore et al. (1997) and McGlothlin et al. (2010), and assuming that the covariance terms in Equation A8 are either zero or population parameters that do not have a heritable component, an individual's total breeding value can be written as the genetic contribution to the population mean

(A12) $$A=\left(1+\psi_{g}\overset{=}{s}\right)a.$$

Equation A12 applies both to populations that consist of single social networks and populations split into groups of equal size. This can also be represented as a sum of a direct breeding value (*a*) and a social breeding value ($\psi_{g}\overset{=}{s}a$). Following McGlothlin et al. (2010), we can now calculate the response to both selections using the Price equation:

(A13) $$\Delta \overset{=}{z}=\text{Cov}\left[A,w\right],$$

where *w* is relative fitness. First, we ignore social selection and model relative fitness as a function of the focal individual's phenotype,

(A14) w=α+βz+ε,

where α is an intercept, β represents individual (nonsocial) selection, and ε is an error term. This gives

(A15) $$\Delta \overset{=}{z}=\text{Cov}\left[A,z\right]\beta$$

and substituting for *z*,

(A16) $$\Delta \overset{=}{z}=\left(1+\psi_{g}\overset{=}{s}\right)\text{Cov}\left[a,a+e+\psi_{g}\left(\bar{sa^{\prime }}+\bar{se^{\prime }}\right)\right]\beta .$$

Making the further assumption that genetic terms are uncorrelated with both environmental terms and connection strengths,

(A17) $$\Delta \overset{=}{z}=\left(1+\psi_{g}\overset{=}{s}\right)G\beta ,$$

where *G* is equal to Var[*a*]. This shows that when interactions occur at random, the amount of genetic variation available for a response to selection should increase as $\psi_{g}\overset{=}{s}$ becomes more positive and decrease as $\psi_{g}\overset{=}{s}$ becomes more negative.

We can relax the assumption that interactions occur at random by allowing individuals to associate with like (homophily) or unlike individuals (heterophily). In this scenario,

(A18) $$\text{Cov}\left[a,\psi_{g}\left(\overset{}{\bar{sa^{\prime }}}+\overset{}{\bar{se^{\prime }}}\right)\right]\neq 0.$$

One reasonable model for this covariance is

(A19) $$\text{Cov}\left[a,\psi_{g}\left(\overset{}{\bar{sa^{\prime }}}+\overset{}{\bar{se^{\prime }}}\right)\right]\approx R\psi_{g}G,$$

where *R* is the regression of $\bar{sa^{\prime }}$ on *a* and is thus analogous to relatedness. This assumes that $\text{Cov}[a,\bar{se^{\prime }}]$ is negligible. Positive values of *R* indicate homophily, and negative values indicate heterophily. Making this substitution gives Equation 4 in the text. Equation 4 can also be expressed differently by rearranging as:

(A20) $$\Delta \overset{=}{z}=\left[G+\;\left(1+R\right)\psi_{g}\overset{=}{s}G+R\psi_{g}^{2}\overset{=}{s}G\right]\beta .$$

Here, *G* represents the variance in direct breeding values and $\psi_{g}\overset{=}{s}G$ represents the covariance between direct and social breeding values. The term $\psi_{g}^{2}\overset{=}{s}G$ is proportional to the variance in social breeding values ($\psi_{g}^{2}\overset{=}{s}{}^{2}G$). Although we ignore multilevel selection social here, we note that indirect genetic effects will magnify among‐group genetic variance (Equation A11), which should contribute to a response to group (or social) selection.
