# Supplementary material for: Social structure modulates the evolutionary consequences of social plasticity: A social network perspective on interacting phenotypes
Source: Ecol Evol. 2017 Dec 27;8(3):1451–64. doi: 10.1002/ece3.3753 (PMC5792542; doi:10.1002/ece3.3753)

**Supplementary material S1: code used to conduct simulations and generate the figures.**


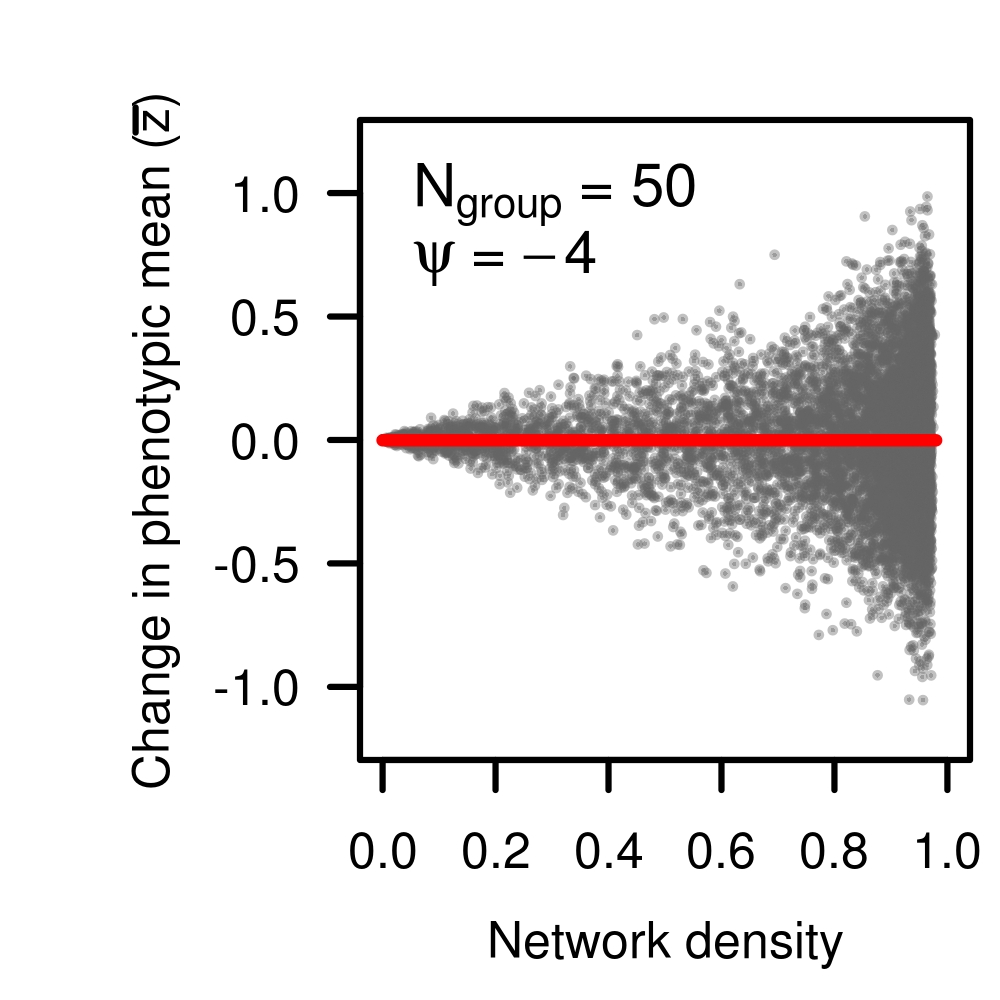
**Supplementary material S2:** The average phenotype of individuals in a social network as a function of network density when ψ is negative. Each dot represents a simulated network, or group of 50 individuals (N_group_ = 50). Indirect genetic effects were generated using a ψ of - 4.

# Supplementary material S3: The average phenotype of individuals in a social network as a function of network density when breeding values are distributed normally. Each dot represents a simulated network, or group of 50 individuals (N_group_ = 50). Indirect genetic effects were generated using a ψ of 4. Breeding values were taken for a normal distribution with a mean of 0 and a variance of 0.3.


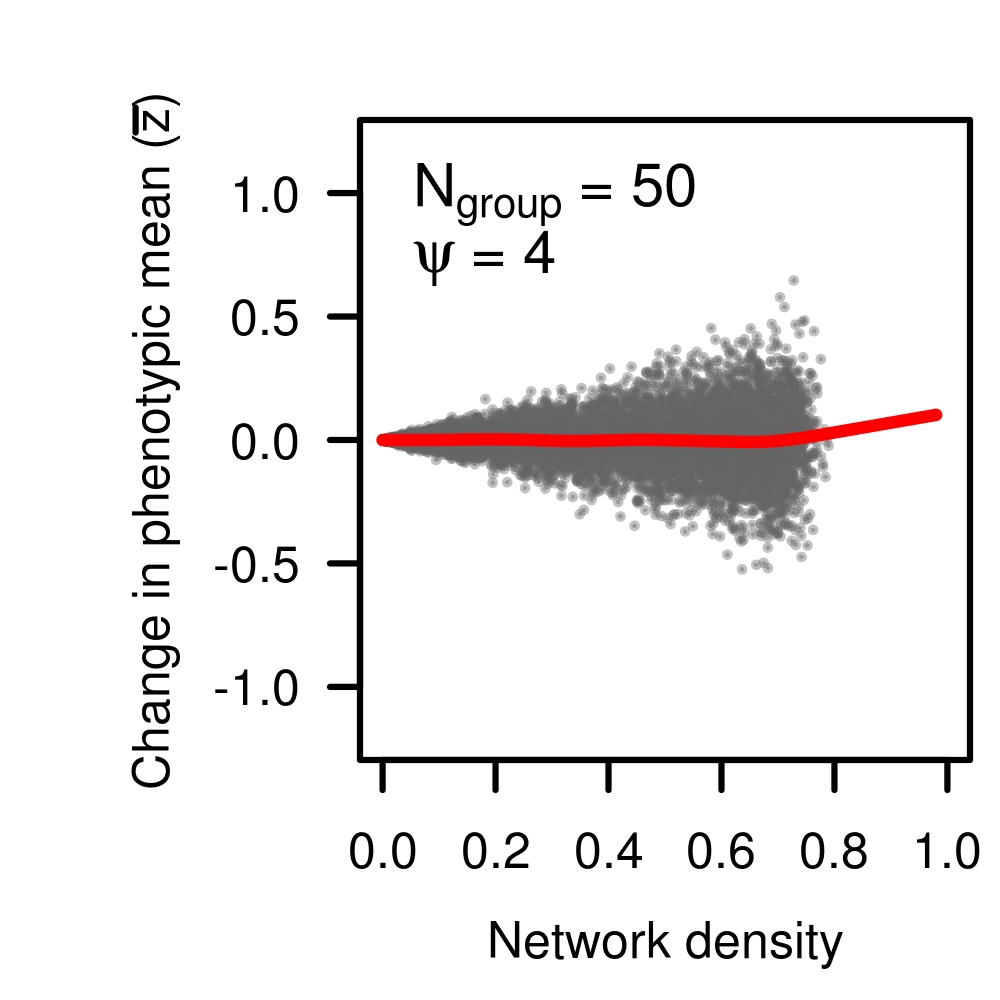


#
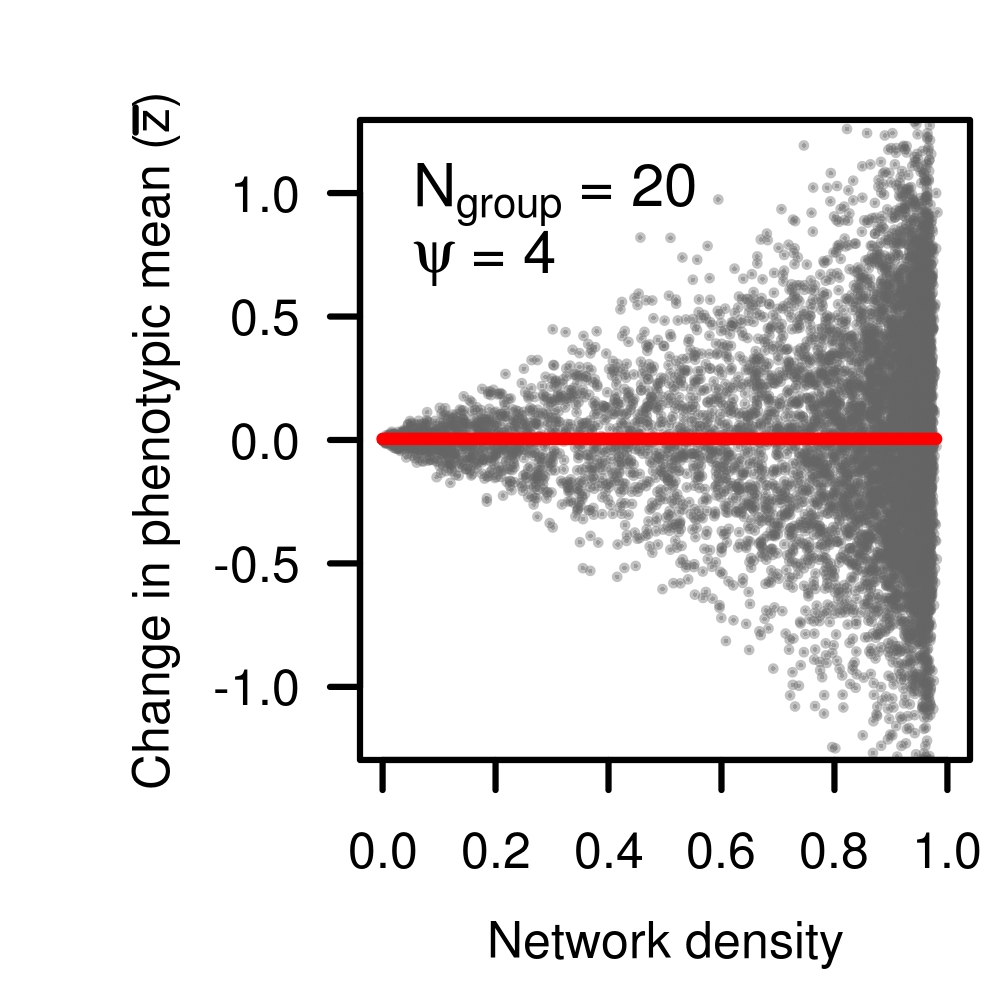
Supplementary material S4: The average phenotype of individuals in a social network as a function of network density in smaller groups. Each dot represents a simulated network, or group of 20 individuals (N_group_ = 20). Indirect genetic effects were generated using a ψ of 4.

# Supplementary material S5: Effects of network density on the correlation between direct and indirect genetic effects in smaller groups. Each dot represents a simulated network, or group of 20 individuals (N_group_ = 20). Indirect genetic effects were generated using a ψ of 4.


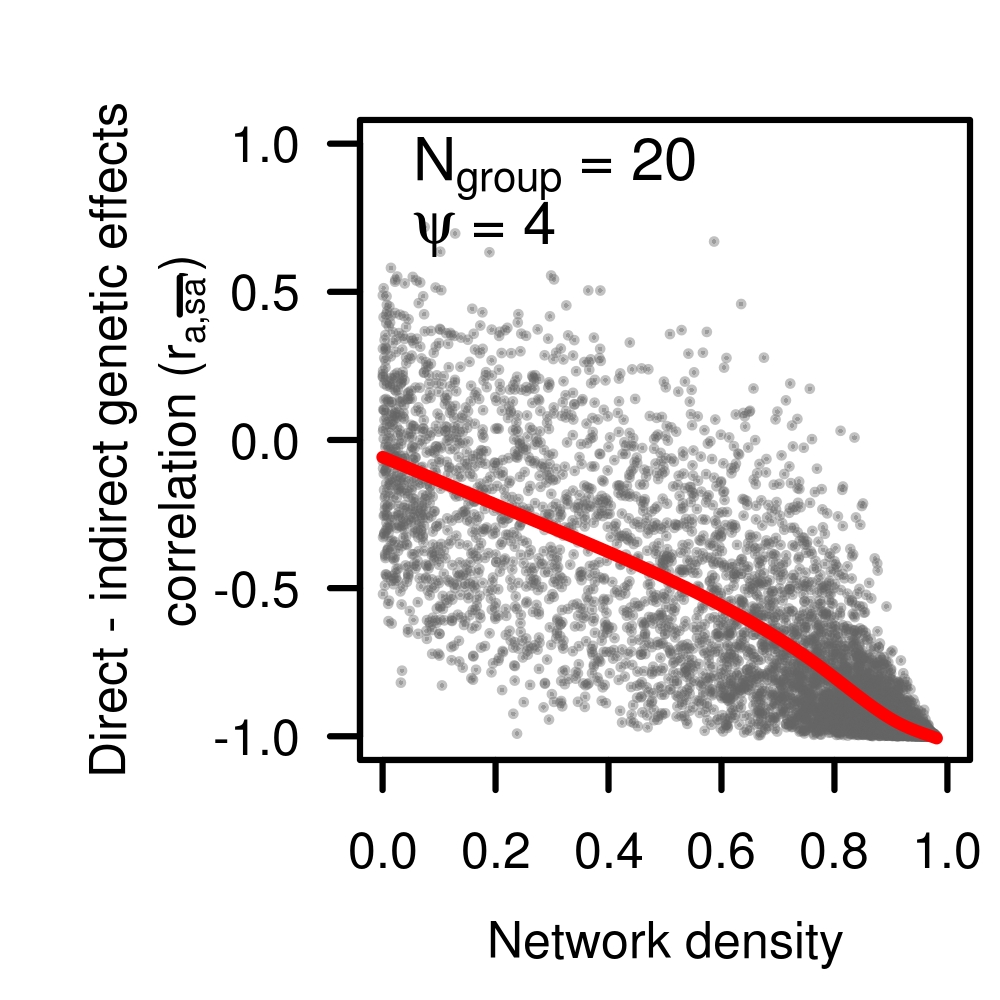


# Supplementary material S6: Effects of network density on the relative change in phenotypic variance within groups. Each dot represents a simulated network, or group of 50 individuals (N_group_ = 50). Indirect genetic effects were generated using a $\boldsymbol{\psi}_{\boldsymbol{g}}$ of - 4.

#
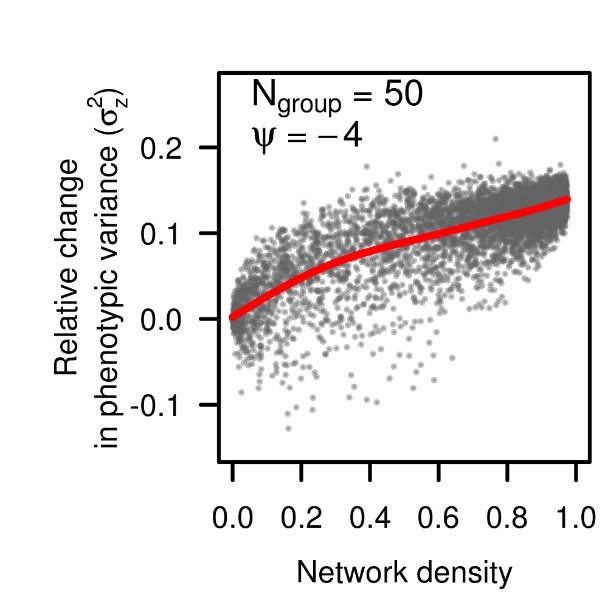


# Supplementary material S7: Effect of network homophily on the relative change in phenotypic variance within groups when ψ is negative. Each dot represents a simulated network, or group of 50 individuals (N_group_ = 50). Indirect genetic effects were generated using a ψ of - 4.


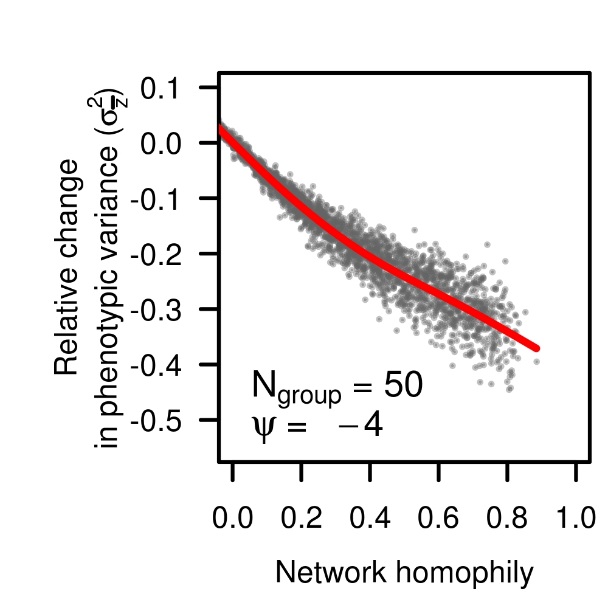

Supplement: Supplementary file 1 [file ECE3-8-1451-s001.docx]
